# The relationships among nutrition, body composition, muscle strength and physical performance in patients with acromegaly

**DOI:** 10.20945/2359-4292-2026-0048

**Published:** 2026-05-15

**Authors:** Natália Nachbar Hupalowski, Claudia Pinheiro Sanches Rocha, Vicente F. C. Andrade, Cesar Luiz Boguszewski, Victoria Zeghbi Cochenski Borba

**Affiliations:** 1 Serviço de Endocrinologia e Metabologia (SEMPR), Hospital de Clínicas, Departamento de Medicina Interna, Universidade Federal do Paraná, Curitiba, PR, Brasil

**Keywords:** Acromegaly, nutrients intake, body composition, physical performance, muscle mass

## Abstract

**Objective:**

To investigate the impact of dietary intake profile on body composition, physical performance, and muscle strength in patients with acromegaly.

**Subjects and methods:**

Observational, cross-sectional study involving patients with acromegaly compared with age and sex-matched controls. Body composition, including total lean mass (TLM) and appendicular lean mass (ALM), was assessed using dual-energy X-ray absorptiometry (DXA). All participants completed a Food Frequency Questionnaire (FFQ) and underwent strength and performance testing.

**Results:**

A total of 82 patients were included, 41 in the acromegaly group (AG) and 41 in the control group (CG). The AG comprised 23 women and 18 men, mean age 55.9 ± 11.8 years and mean BMI 31.1 ± 5.2 kg/m^2^. Muscle mass was increased in the AG, but strength and physical performance were worse compared to CG. AG exhibited a higher intake of carbohydrates, trans fats, and certain micronutrients, as omega-3, vitamin B9, and beta-carotene, compared to the CG. Niacin (R = -0.316, p = 0.004) and vitamin B6 (R = -0.320, p = 0.042) were associated with performance on the sit to stand test. Beta-carotene (R=-0.429, p=0.005), vitamin E (R = -0.321, p=0.041), and flavone (R = -0.313, p = 0.046) were associated with better time to get up and go (TUG) test performance, whereas caffeine intake (R = 0.344, p = 0.028) was associated with worse time. Additionally, niacin (R = 0.341, p = 0.029) and selenium (R = 0.317, p = 0.046) had a positive impact on the short physical performance battery (SPPB) test. Hand grip strength was positively correlated with monounsaturated fats (R = 0.387, p = 0.012) and selenium (R = 0.316, p = 0.044). Selenium, zinc, omega-6 fatty acids, calcium, and iron, were all positively associated with TLM, while caffeine and isoflavones showed a negative association.

**Conclusion:**

Patients with acromegaly exhibited increased muscle mass, but their functional capacity were compromised, potentially due to alterations in muscle composition. The intake of nutrients such as selenium, β-carotene, flavonoids, vitamin E and niacin, may improve physical performance and muscle strength.

## INTRODUCTION

Acromegaly is a rare endocrine disorder characterized by excessive secretion of growth hormone (GH) and insulin-like growth factor 1 (IGF-1), typically due to a GH-secreting pituitary adenoma. This hormonal excess leads to a range of clinical features ^(1)^, which increase the risk of comorbidities and is accompanied by notable alterations in body composition. Patients with active disease often exhibit a reduction in visceral fat mass and an increase in skeletal muscle mass and extracellular body water ^(1)^. Although increased muscle mass may suggest better physical capacity, elevated GH and IGF-I levels can negatively impact peripheral muscle strength, exacerbating musculoskeletal weakness ^(2)^.

Muscle health maintenance relies on essential nutrients. Proteins and amino acids play a central role in preserving and increasing muscle mass by stimulating muscle protein synthesis. Studies indicate that adequate protein intake combined with resistance training is crucial for promoting muscle anabolism ^(3)^. Furthermore, higher intake of proteins and dietary fiber has been associated with lower body fat percentage and body mass index (BMI), demonstrating benefits for body composition and fat control ^(4)^. Polyunsaturated fatty acids, such as omega-3, known for their anti-inflammatory properties, also contribute to increased muscle mass and strength by modulating anabolic signaling pathways ^(5)^. These lipids are further associated with reduced central adiposity, particularly when combined with strength training ^(5)^.

Antioxidant vitamins (C and E) play a crucial role in protecting muscle tissue from oxidative stress, thereby supporting muscle health ^(6,7)^. In addition, other nutrients such as iron, niacin (vitamin B3), zinc, vitamin B12, selenium, and proteins are beneficial to lean mass ^(8)^. Experimental and observational studies suggest that adequate selenium levels are associated with a lower body fat percentage and a better lean mass profile, whereas both deficiency and excess may impair adipose tissue function ^(9,10)^. Monounsaturated fats intake is inversely associated with the risk of sarcopenia, suggesting a protective role in the preservation of muscle mass ^(11)^. Additionally, dietary patterns that promote the intake of these fats may improve strength, muscle power, and antioxidant markers ^(12)^. While some nutrients demonstrate beneficial effects on body composition and muscle strength, others may exert negative impacts. Trans fats, for instance, are associated with detrimental effects on skeletal muscle mass ^(13)^. These lipids impair insulin sensitivity in muscle,interfering with energy metabolism and potentially contributing to a progressive decline in muscle strength and function ^(14,15)^. This effect may exacerbate insulin resistance, to which individuals with acromegaly are more susceptible due to the excess of GH and IGF-1 ^(16)^.

Chronic alcohol consumption is also linked to a reduction in muscle mass and strength, largely attributed to the inhibition of protein synthesis and the increased rate of muscle degradation ^(17,18)^.

Given the limited literature on the nutritional intake of patients with acromegaly and its effects on body composition, physical performance, and muscle strength, this study aims to evaluate the influence of dietary profile on these parameters.

## SUBJECTS AND METHODS

This observational, cross-sectional study evaluated patients with acromegaly who were followed at the Neuroendocrine Unit of the Endocrine Division (SEMPR) in our academic research center. The study was approved by the Ethics Committee of our institution under the protocol number 6.293.233 (CAAE: 49629321.7.0000.0096), and all individuals who agreed to participate signed an informed consent form.

Patients with acromegaly of both sexes over the age of 18 were invited to participate by convenience sampling during their routine outpatient medical appointment. The exclusion criteria included uncontrolled metabolic or chronic diseases (e.g., uncontrolled diabetes, uncontrolled hypertension, chronic kidney disease) or active malignant neoplasms; use of medications or drugs that could affect muscle or bone (e.g., hormones, corticosteroids, and GLP-1 agonists); any physical impairment preventing physical function assessment; and patients who were unable to perform the required tests and exams.

The included patients with acromegaly comprised the acromegaly group (AG) and completed a standardized questionnaire about demographic data, disease characteristics, type of treatment, medications in use, lifestyle habits (smoking and alcohol consumption), personal and family history of fractures, and the presence of comorbidities. Data not captured during the interview were collected from medical records. The participants were then referred for nutritional and physical evaluations (weight, height, and body mass index [BMI]), body composition assessment, strength and functional tests.

The diagnosis of acromegaly was based on the 14th Acromegaly Consensus, and the AG was considered to have controlled disease if the IGF-1 levels were within the normal reference range for age and sex ^(19)^. A control group (CG), matched by sex and age, was recruited from relatives of patients and individuals from the community.

### Body composition

Body composition was assessed using dual-energy X-ray absorptiometry (DXA) with a Hologic Horizon A device - serial number 201383, Bedford, USA. Parameters analyzed included total lean mass in grams (TLM), percentage of total fat mass (%TFM), android/gynoid ratio (A/G), and appendicular lean mass (ALM), measured by the sum of lean mass in the arms and legs. Weight was measured using a calibrated anthropometric scale (Plenna^®)^ with the participant barefoot. Height was measured using a vertical stadiometer (Tonelli Gomes^®)^. Body mass index (BMI) was calculated by dividing weight (in kg) by height squared (in meters). BMI results for participants were classified as underweight (BMI < 18.5 kg/m^2)^, normal weight (18.6 kg/m^2^ < BMI < 24.9 kg/m^2)^, overweight (25.0 kg/m^2^ < BMI < 29.9 kg/m^2)^, obesity grade I (30.0 kg/m^2^ < BMI < 34.9 kg/m^2)^, obesity grade II (35 kg/m^2^ < BMI < 39.9 kg/m^2)^, and grade III obesity (BMI > 40 kg/m^2)^ ^(20)^.

### Physical performance and muscle strength

Physical performance and strength were evaluated using the short physical performance battery (SPPB), which comprises three tests: strength, performance, and balance. Strength was assessed through the 5-times seat to stand test (STST), where patients were required to rise from and sit back on a chair five consecutive times as quickly as possible without stopping or using their arms. A time below 15 seconds (s) was considered normal ^(21)^. Performance was measured using the 4-meter gait speed test (GS), in which patients stood up from a chair, walked 6 meters at a safe speed, the first and last meters were not timed; a speed ≥0.8 m/s was considered normal ^(22)^. Balance was evaluated by instructing patients to maintain a standing position without support for 10s with their feet together, followed by another 10s in a semi-tandem position, and then 10s in a tandem position. Each position was scored: maintaining the position for 10s earned 2 points, holding it for 3 to 9.99s earned 1 point, and less than 3s or inability to perform resulted in 0 points ^(22)^. Additionally, the time up and go test (TUG) was conducted, requiring patients to stand from a chair without using their arms, walk 3 meters, turn around a cone, return, and sit back down at a comfortable pace. A time of 20s or less was considered normal ^(22,23)^. Upper limb strength was assessed using the handgrip strength (HGS) test with a Charder^®^ MG 4800 dynamometer. Patients were instructed to squeeze the dynamometer handle with maximum strength, and an average of three attempts was recorded. Values of ≤ 27 kg for men and ≤16 kg for women were considered altered ^(22)^.

### Food Frequency Questionnaire (FFQ)

To assess dietary patterns and nutrient intake, a validated food frequency questionnaire (FFQ) was used for the adult Brazilian population aged 18 to 60 years, who were overweight, i.e., BMI above 25 kg/m^2^ ^(24)^. The FFQ was collected considering the foods consumed over a one-month period and includes 90 foods or preparations, divided into fifteen sections: 1) foods or preparations; 2) pasta; 3) various dishes and snacks; 4) meats; 5) rice, tubers, and vegetables; 6) eggs; 7) dairy products; 8) fats; 9) cereals; 10) green leaves; 11) fruits and juices; 12) miscellaneous; 13) breads, cakes, and cookies; 14) various drinks; and 15) sweets and desserts. During the interview, the participant was asked to indicate whether they consumed the food or preparation, the frequency throughout the month, and the amount consumed in household measures or the approximate value in grams.

During the face-to-face interview in which the food frequency questionnaire was administered, the research team used a standardized photographic manual containing household utensils and portion-size images with corresponding weights ^(25)^. Participants were asked to identify the portion size that most closely resembled their usual intake. These standardized images were not photographs of the participants’ own meals, but visual aids used to improve accuracy in estimating portion sizes. FFQ data were entered into a pre-designed Excel^®^ spreadsheet, which displayed the quantities of 38 macronutrients, micronutrients, and bioactive compounds per 100 grams of food or preparation. A formula was created, so when the quantity consumed by the patient was entered into the spreadsheet, the calculation was automatic. The calculation of dietary component intake used the Brazilian food composition table (TACO) ^(26)^, the USDA food and nutrient database ^(27)^, the Brazilian table of carotenoid composition in foods ^(28)^, and the Phenol-Explorer 3.0 database ^(29)^. The intake pattern was considered inadequate when nutrient intake was below the recommended levels for age and sex, based on Dietary Reference Intakes (DRIs). Thus, the Estimated Average Requirement (EAR) was used, and if EAR values were not available, the Adequate Intake (AI) ^(30)^, and the latest WHO guidelines on fat and carbohydrate consumption were applied ^(31,32)^.

### Statistical analysis

Statistical analyses were performed using the Statistical Package for the Social Sciences (SPSS) software, version 29. A descriptive analysis of the frequency distribution of all variables was conducted. The Kolmogorov-Smirnov test was used to assess the normality of the variables in the study. To evaluate differences between means, Student’s t-test was used for parametric data and the Mann-Whitney test for non-parametric data. For the evaluation of categorical data, Fisher’s exact test and the chi-square test were used. We employed the Spearman’s Rho coefficient to measure the size of the effect of the studied associations, assuming the following cutoff points: at least 0.8 (very strong), 0.6 up to 0.8 (moderately strong), 0.3-0.5 (fair), and < 0.3 (poor) ^(33)^. Statistical significances were considered when the p-value was ≤ 0.05.

## RESULTS

Fifty-seven patients with acromegaly were invited to participate, eight were excluded either because they lived outside town and could not do the evaluations or had a condition that could impact the test results. Additionally, eight individuals declined to answer the FFQ. Finally, 41 patients were included in the AG (mean age 55.9 ± 11.8 years; 90.2% white and 58.5% women; BMI 31.3 ± 5.2 kg/m^2)^ and were compared to 41 in the CG (mean age 56.8 ± 14.3 years; 82.9% white and 56.1% women; BMI 25.5 ± 3.3 kg/m^2)^. The mean age at diagnosis of acromegaly was 43.7 ± 13.0 years, and the mean disease duration was 12 years. Most of the patients in the AG underwent surgical treatment.

Compared to the CG, the AG group had higher BMI, more fractures, and a higher number of comorbidities, with hypertension, obesity, and hypogonadism being the most prevalent (p < 0.05 for all) (**Table 1**).

**Table 1:** Clinical and nutritional characteristics of the Acromegaly Group (AG) and the Control Group (CG)

Characteristics	AG (n = 41)	CG (n = 41)	P
Age (years)	55.9 ± 11.8	56.8 ± 14.3	0.769
Ethnicity			0.01
White, n (%)	38 (92.7)	34 (82.9)	
Black, n (%)	3 (7.3)	1 (2.4)	
Asian, n (%)	0	6 (14.6)	
Sex, n (%)			
Female	23 (56.1)	23 (56.1)	
Male	18 (43.9)	18 (43.9)	
Smoking, n (%)	7 (17.1)	1 (2.4)	0.02
Height (m)	1.70 ± 0.1	1.64 ± 0.1	0.445
Weight (kg)	87.0 ± 18.6	70.1 ± 11.3	< 0.001
BMI (kg/m^2)^	31.1 ± 5.2	25.5 ± 3.3	< 0.001
Normal, n (%)	6 (14.6)	25 (61.0)	
Overweight, n (%)	15 (36.6)	11 (26.8)	
Obesity grade I, n (%)	10 (24.4)	5 (12.2)	
Obesity grade II, n (%)	7 (17.1)	0	
Obesity grade III, n (%)	3 (7.3)	0	
Disease data			
Time since diagnosis (years)	12.0 ± 7.8	NA	
Age at diagnosis (years)	43.7 ± 13.0	NA	
Serum IGF-1^*^ (ng/mL)	256.6 ± 164.8	NA	
Surgical treatment, n (%)	35 (85.4)	NA	
Disease control, n (%)			
Controlled	26 (63.4)	NA	
Uncontrolled	15 (36.6)	NA	
Medications, n (%)			
Somatostatin receptor ligands			
Octreotide	26 (63.4)	NA	
Lanreotide	15 (36.6)	NA	
Cabergoline	7 (17.1)	NA	
Pegvisomant	4 (9.7)	NA	
Comorbidities, n (%)			
Hypertension	23 (56.1)	14 (34.1)	0.057
Obesity	20 (48.8)	6 (14.6)	0.001
Dyslipidemia	16 (39.0)	12 (29.3)	0.390
Diabetes	15 (36.6)	3 (7.3)	0.003
Hypothyroidism	9 (21.9)	1 (2.4)	0.01
Hypogonadism	9 (21.9)	0	< 0.001
Men	4 (44.4)	0	
Hormonal replacement	1 (25.0)	0	
Women	5 (55.5)	0	
Hormonal replacement	4 (80.0)	0	
Hypopituitarism	7 (17.1)	0	0.01
Mood disorder	6 (14.6)	0	0.032
Osteoporosis	5 (12.2)	4 (9.7)	1.000
Fracture/patient	0.63 ± 1.11	0.15 ± 0.43	0.01

NA: not applicable; BMI: body mass index;

* value of IGF-1 at the time of evaluation.

Nutritional inadequacies were common in both groups, despite differences in the intake of specific nutrients and bioactive compounds (**Table 2**). The AG had higher intake of carbohydrates (p = 0.019), trans fats (p < 0.001), omega-3 (p = 0.001), folic acid (p = 0.013), thiamine (p = 0.009), and beta-carotene (p 0.001). The mean mineral intake was similar between groups. Both groups showed insufficient daily consumption of fiber, omega-3, omega-6, vitamins A and E, magnesium, potassium, and calcium; only the CG had folic acid intake below the recommended level.

**Table 2:** Nutrient intake in the Acromegaly Group (AG) and the Control Group (CG)

Nutrients	EAR	AG (n = 41) Mean ± SD	CG (n = 41) Mean ± SD	*p*
Energy (kcal)		2242.34 ± 929.36	1958.42 ± 784.35	0.059
Macronutrients				
Proteins (g)		104.15 ± 45.13	104.13 ± 48.12	0.893
Carbohydrates (g)	100	267.78 ± 130.47	207.88 ± 96.50	0.019
Fibers (g)	25-38	19.20 ± 7.50	16.78 ± 7.63	0.084
Total fat (g)	ND	71.86 ± 32.73	69.69 ± 33.47	0.640
Saturated (g)	< 10%/day	25.58 ± 12.16	25.13 ± 10.53	0.835
Monounsaturated (g)	ND	23.59 ± 11.12	23.38 ± 12.16	0.738
Poliunsaturated (g)	ND	12.82 ± 5.86	12.26 ± 5.36	0.745
Cholesterol (mg)	<300	383.79 ± 179.79	426.79 ± 212.00	0.325
Trans fat (g)	< 1%/day	2.86 ± 2.55	0.76 ± 0.60	< 0.001
Omega-3 (g)	1.1-1.6	0.36 ± 0.24	0.21 ± 0.18	0.001
Omega-6 (g)	11-17	9.86 ± 5.02	9.15 ± 6.87	0.594
Micronutrients				
Vitamins				
Vitamin A (mg)	500-625	174.07 ± 113.96	174.82 ± 96.15	0.974
Vitamin C (mg)	60-75	188. 73 ± 136.66	145.48 ± 106.18	0.068
Vitamin D (UI)	400UI	182.4 ± 185.2	182 ± 205.6	0.670
Vitamin E (mg)	12	3.86 ± 2.32	4.56 ± 2.66	0.208
B1 (mg)	0.9-1.0	1.58 ± 0.67	1.23 ± 0.51	0.009
B3 (mg)	14-16	25.2 ± 12.2	23.6 ± 11.9	0.530
B6 (mg)	1.1-1.3	1.96 ± 0.97	1.83 ± 0.82	0.503
B9 (mcg)	320	460.42 ± 171.09	371.86 ± 171.96	0.013
B12 (mcg)	2.0	4.44 ± 2.26	4.76 ± 2.42	0.467
Minerals				
Calcium (mg)	800-1000	636.08 ± 380.79	583.27 ± 281.71	0.935
Magnesium (mg)	255-350	280.13 ± 90.54	259.91 ± 98.59	0.336
Potassium (mg)	2600-3400	3056.63 ± 1144.71	2762.02 ± 0.964	0.272
Zinc (mg)	6.8-9.4	12.79 ± 6.57	12.26 ± 5.36	0.802
Trace element				
Selenium (mcg)	45	101.91 ± 43.08	104.02 ± 44.48	0.828
Bioactive compounds				
Betacarotene (mcg)	ND	810.71 ± 592.22	252.65 ± 347.75	0.001
Flavone (mg)	ND	9.33 ± 5.27	5.27 ± 7.70	0.153
Isoflavone (mg)	ND	0.68 ± 0.71	0.66 ± 0.65	0.893
Others				
Alcohol (g)	< 30	1.31 ± 3.66	9.10 ± 11.43	< 0.001
Caffeine (g)	400	84.48 ± 71.85	108.63 ± 107.04	0.323

EAR: estimated average requirement; B12: methylcobalamin; B6: pyridoxine; B9: folic acid; B1: thiamine; B3: niacin.

The body composition showed similar %TFM and A/G ratio between groups. In contrast, lean mass were higher in the AG, TLM (male AG 66.7 ± 8.9 kg vs CG 52.3 ± 8.6 kg, p < 0.001 and female AG 47.1 ± 9.1 kg vs CG 38.7 ± 6.0 kg, p = 0.001) and ALM in both men and women (male AG 27.7 ± 4.3 kg vs CG 22.3 ± 3.1 kg, p < 0.001 and female AG 16.9 ± 6.4 kg vs CG 15.0 ± 2.4 kg, p = 0.009) (**Table 3**).

**Table 3:** Body Composition in the Acromegaly Group (AG) and the Control Group (CG)

	AG (n = 41)	CG (n = 41)	p
Total lean mass (kg)			
Men	66.7 ± 8.9	52.3 ± 8.6	< 0.001
Women	47.1 ± 9.1	38.7 ± 6.0	0.001
Total fat mass (%)			
Men	32.0 ± 3.5	31.2 ± 4.0	0.528
Women	41.3 ± 5.6	42.0 ± 3.7	0.644
ALM (kg)			
Men	27.7 ± 4.3	22.3 ± 3.1	<0.001
Women	16.9 ± 6.4	15.0 ± 2.4	0.009
A/G ratio			
Men	1.11 ± 0.17	1.07 ± 0.14	0.445
Women	0.89 ± 0.11	0.91 ± 0.12	0.543
Elevated A/G ratio, n (%)			
Men	14 (87.5)	17 (89.5)	0.855
Women	13 (59.1)	13 (68.4)	0.536

ALM: appendicular lean mass; A/G: android/gynoid ratio.

Data on physical performance and muscle strength are presented in **Table 4**. Compared to the CG, the AG showed slower STST (16.6 s ± 5.3 s vs 12.2 s ± 2.5 s; p < 0.001), TUG (12.22 s ± 4.27 s vs 9.4 s ± 2.7 s; p < 0.001) and GS (0.91 m/s ± 0.3 m/s vs 1.16 m/s ± 0.2 m/s; p < 0.001), and lower SPPB scores (9.0 ± 2.6 vs CG 11.8 ± 0.5; p < 0.001).

**Table 4:** Physical performance and muscle strength in the Acromegaly Group (AG) and the Control Group (CG)

Tests	AG (n = 41)	CG (n = 41)	p
Muscle strength STST (s)	16.6 ± 5.3	12.2 ± 2.5	<0.001
Low n (%)	22 (53.6)	2 (4.9)	<0.001
HGS (kg) Men	42.1 ± 9.3 (0)	39.4 ± 5.8 (0)	0.293
Low n (%)	0 (0)	0 (0)	
Women	25.7 ± 5.8 ^(1)^	24.6 ± 4.7 (0)	0.488
Low n (%)	1 (2.43)	0 (0)	1.000
Physical performance TUG (s)	12.22 ± 4.27	9.4 ± 2.7	0.001
Altered	3 (7.3)	1 (2.4)	0.616
GS (m/s)	0.91 ± 0.3	1.16 ± 0.2	< 0.001
Altered	12 (29.3)	3 (7.3)	0.020
SPPB	9.0 ± 2.6	11.8 ± 0.5	< 0.001
Altered	13 (31.7)	0 (0)	< 0.001

AG: acromegaly group; CG: control group; n: number; %: percentage; s:seconds; kg: kilograms; m/s: meters per second; STST: 5-times seat to stand test; HGS: hand grip strength; TUG: time up and go test; GS: gait speed; SPPB: short physical performance battery test.

**Figure 1:**
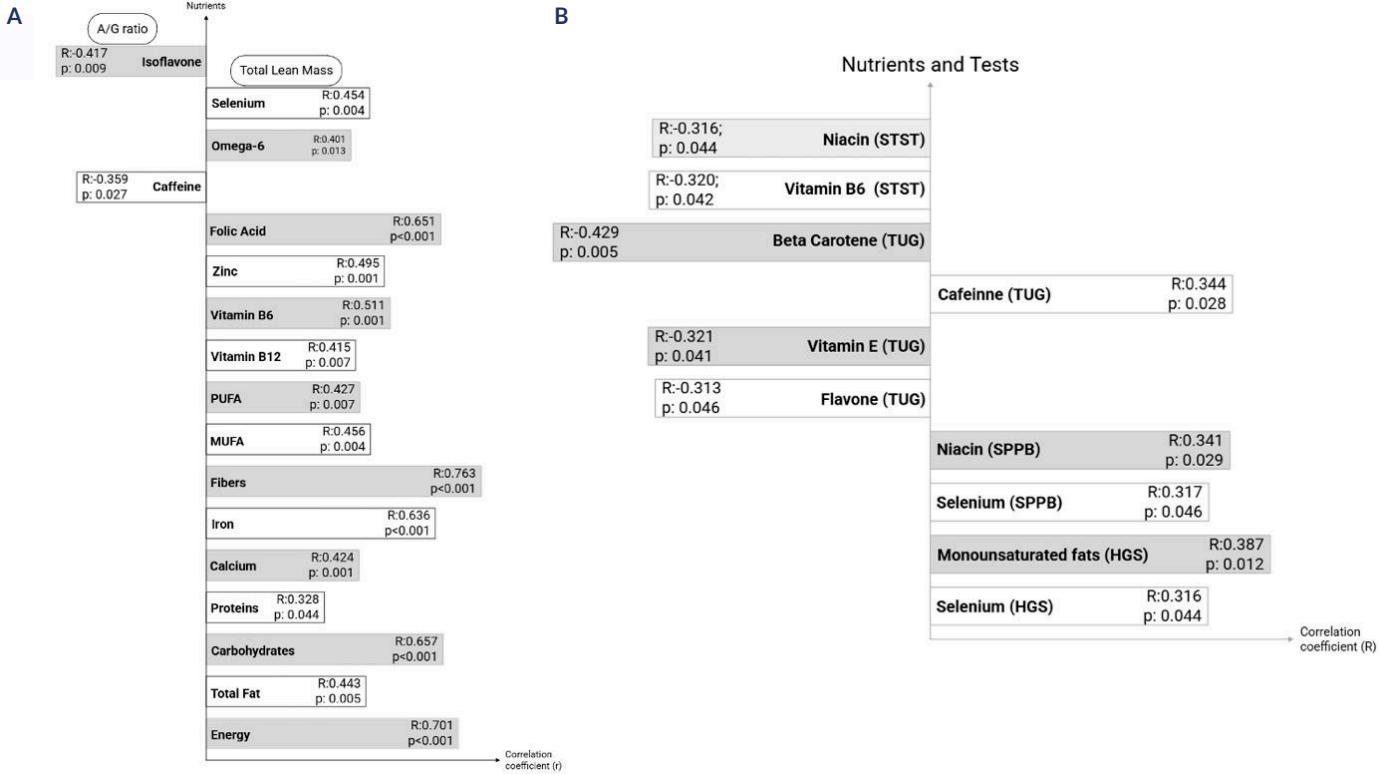
Correlation between nutrients with body composition (**A**), muscle strength and physical performance (**B**).

PUFA: polyunsaturated fats; MUFA: monounsaturated fats; TLM: total lean mass; A/G: android/gynoid ratio; TUG: time up and go test; STST: 5- times sit-to-stand test; HGS: handgrip strength; SPPB: Short Physical Performance Battery.

Correlations between body composition, physical performance tests, muscle strength and the intake of nutrients and bioactive compounds in the AG are detailed in **Figure 1**. The A/G correlated negatively only with isoflavone, while TLM correlated positively with several nutrients, including selenium, omega-6, folic acid, zinc, vitamins B6 and B12, polyunsaturated fats (PUFA), monounsaturated (MUFA), iron, fiber, calcium, proteins, carbohydrates, total fat, and total calories (p < 0.05 for all). Only caffeine showed a negative correlation with TLM (**Figure 1A**).

Higher intake of beta-carotene, flavonoids, and vitamin E was associated with shorter TUG time, whereas caffeine intake was associated with longer TUG time. Higher intake of monounsaturated fats and selenium was positively correlated with HGS, whereas higher niacin and vitamin B6 intake was inversely correlated with STST time (**Figure 1B**).

Although higher ALM and TLM were observed in patients with uncontrolled disease (ALM 25.16 ± 5.6 kg uncontrolled vs. 19.25 ± 8.0 kg controlled, p = 0.028 and TLM 61.5 ± 12.5 kg uncontrolled vs. 51.3 ± 12.5 kg controlled, p = 0.019). No differences in nutrient intake were observed between patients with controlled versus uncontrolled acromegaly.

## DISCUSSION

To our knowledge, this is the first study to evaluate dietary quality in patients with acromegaly and examine its association with body composition, physical performance and muscle strength. Nutritional intake differed between patients and controls and inadequacies in several nutrients and bioactive compounds were common in both groups. Notably, the AG showed higher TLM but poorer strength and physical performance associated with modifications in diet quality, suggesting that increased lean mass does not necessarily translate into better function in this population.

Patients with acromegaly presented significantly higher total body weight compared to the CG largely driven by lean mass. The higher TLM in the AG could be attributed to the anabolic role of GH and IGF-1 in promoting protein synthesis. Previous studies support the altered body composition in acromegaly, characterized by increased skeletal muscle mass and reduced body fat ^(34,35)^. However, in our sample, there was no significant difference in body fat percentage or A/G between groups, suggesting that the higher body weight in the AG was predominantly due to increased lean mass, which may also reflect an increase in intramuscular water. TLM was also associated with the intake of various nutrients, including vitamins (B6, B9, and B12), minerals (iron, zinc, calcium, and selenium) and macronutrients (carbohydrates, proteins, and total fats), suggesting that the diet may modulate lean-mass measures in this group.

It was observed that, despite having greater lean mass, patients with acromegaly exhibited a poorer physical function, indicating muscle weakness, as previously reported ^(36)^. This deficit was evident in the results of physical performance tests, where individuals with acromegaly performed worse compared to the CG. This altered functionality may be related to increased collagen deposition in muscle tissues, impairing flexibility, and muscle efficiency ^(37)^. A study by Martel-Duguech and cols. found that patients with acromegaly had slower gait speed and poorer performance in the STST ^(36)^, corroborating our findings. Several nutrients have been positively correlated with better physical performance and body composition, and a balanced, nutrient-rich diet is generally associated with better performance than a nutrient poor diet ^(38,39)^.

The positive association between vitamin B6 intake and improved performance has been previously demonstrated ^(40)^. However, lower intakes of carbohydrates, omega-3, vitamin D, and saturated fats have been previously observed in individuals with greater frailty ^(41)^. Although the AG had higher carbohydrate and omega-3 intakes than the CG, this did not translate into better muscle strength or performance. Saturated fat intake, despite being similar between groups, exceeded recommendations in both groups. In a study involving women, aged 18-79 years, saturated fat intake was inversely associated with lean mass ^(13)^, whereas in another study of sedentary men aged 25-45 years, a high-fat diet was associated with reduced whole-body efficiency ^(42)^. In our study, mean HGS was similar between the groups, but was better with higher selenium and MUFA intake. A study using data from the National Health and Nutrition Examination Survey (NHANES 2011-2014) higher selenium concentrations were associated with better HGS ^(43)^.

TLM was positively associated with several nutrients, including minerals, vitamins, carbohydrates (including fiber), and fat. These findings suggest that a higher intake of these nutrients may be associated with higher lean-mass measures in these individuals. In the study conducted by Borda and cols. ^(8)^ with older adults, ALM was positively correlated with PUFAs, zinc, selenium, vitamin B12, niacin, vitamin D, iron, and proteins intake, highlighting the potential role of these nutrients in supporting lean mass ^(8)^.

In our study, daily intake of fiber, omega-3, vitamins A, D, and E, magnesium, and calcium, although below the recommended levels for age and sex was positively correlated with body composition and muscle strength. However, because mean intake of these nutrients did not differ significantly between the AG and the CG, we can only hypothesize that meeting recommended daily intakes might be associated with better performance in the AG, potentially mitigating disease-related functional impairment. Alcohol consumption, which is known to negatively impact muscle mass ^(17,18)^ was higher in CG, raising the possibility that it may not represent a significant risk factor in this context, or the increased alcohol intake in CG may have impacted a lower lean mass compared to the AG.

Carbohydrates are the primary energy source for exercise, especially high-intensity activities and play an important role in physical performance and muscle strength. Muscle glycogen is the storage form of carbohydrates, and low glycogen availability can impair physical performance ^(44)^. We observed higher carbohydrate intake in AG, which is likely due to the higher basal metabolism and total energy expenditure in acromegaly ^(45,46)^. Surprisingly, higher carbohydrate intake was not associated with better physical performance, suggesting that other disease related factors may be more important determinants of function.

The folic acid (vitamin B9) is essential for red blood cell production, DNA synthesis, and amino acids ^(33)^. In a two-year study of older adults supplemented simultaneously with vitamin B9 and vitamin B12 showed that the supplementation did not improve physical function ^(47)^. Similarly, in our study, despite higher folic acid intake in AG, it was not associated with physical performance.

Omega-3, an essential fatty acid, must be obtained through diet. Its anti-inflammatory properties are well established. Omega-3 supplementation (2 g or more per day) has been associated with improvements in GS and TUG performance, and modest increases in muscle mass ^(48)^. Omega-3 is a type of PUFA, and in our study, PUFAs were positively correlated with TLM. The intake of omega-3 was assessed from diet rather than supplementation. It remains unclear whether supplementation could potentially improve physical performance and muscle strength in patients with acromegaly ^(48)^.

Trans fat was nearly four times higher in the AG than in the CG. This type of fat is associated with increased all-cause mortality and is known to induce inflammation and oxidative stress ^(49)^. Evidence on trans fats and physical performance largely comes from animal studies. Liou and cols., 2013 reported reduced grip strength in mice fed with a high trans-fat diet ^(50)^. Similarly, Jeyakumar and cols., 2011 reported that trans-fat intake reduced insulin-stimulated glucose uptake in skeletal muscle in female rats, potentially impairing energy production and muscle function ^(14)^. These findings suggest that higher trans-fat consumption in the AG contributed to poorer muscle function, although causality cannot be inferred from our design.

Beta-carotene, a precursor of vitamin A, has been associated with strength and performance. In the Framingham Offspring study, higher total carotenoid intake was associated with increased grip strength and a higher walking speed in adults, suggesting a potential role for antioxidants in preserving muscle function ^(51)^. In our study, beta carotene intake was approximately three times higher in AG than in CG but was not associated with functionality.

Higher concentrations of selenium have been associated with better mobility and less impairment of limb performance, muscle weakness, and mobility in older adults ^(52)^ and with better grip strength in adults without chronic diseases ^(43)^. In our study, selenium intake was positively correlated with HGS, SPPB, and TLM. HGS was similar between groups, which raises the possibility that selenium intake may have contributed to comparable HGS despite overall poorer performance of the AG; however, this interpretation is speculative. Although selenium correlated with SPPB within the AG, mean SPPB was still lower in this group than in the CG, highlighting that multiple factors likely contribute to functional impairment.

Although the AG showed a higher consumption of some nutrients compared to the CG, the FFQ indicated that most participants did not meet recommended daily intake for several micronutrients relevant to health and physical performance. The relatively small sample size and the cross-sectional design limit the generalizability and preclude causal inference. In addition, dietary supplements and medication effects on body composition and muscle strength were not assessed and may have influenced the findings. Dietary intake estimation is also subject to measurement bias. Poorer performance in the AG may reflect balance and gait disturbances, reported in acromegaly rather than intrinsic muscle impairment alone. Finally, groups differed in comorbidities and lifestyle factors - particularly the higher prevalence of diabetes and hypertension in the AG - which may have affected muscle health and physical function. A strength of this study is its focuses on dietary intake in acromegaly and its associations with body composition and muscle functionality, areas that remain underexplored.

In conclusion, our findings highlight the complex relationship between nutritional intake, body composition, strength and physical performance in patients with acromegaly. Despite the increased muscle mass, patients with acromegaly showed poorer physical performance and strength. The higher intake of some nutrients, such as selenium, β-carotene, flavonoids, vitamin E, and niacin, may prove to be potentially beneficial, and future studies, ideally prospective, are needed to clarify whether modification in the diet can improve physical function in patients with acromegaly.

## Data Availability

datasets related to this article will be avail-able upon request to the corresponding author.
